# Supplementary material for: Enrichment of SARM1 alleles encoding variants with constitutively hyperactive NADase in patients with ALS and other motor nerve disorders
Source: eLife. 2021 Nov 19;10:e70905. doi: 10.7554/eLife.70905 (PMC8735862; doi:10.7554/eLife.70905)
Supplement: Figure 8—source data 1. [file elife-70905-fig8-data1.docx]

|  |  |  |  | **Strong GoF** | | | | | | | |  | **Other** | | | | | | | | | | | | | | | |  |  |
| --- | --- | --- | --- | --- | --- | --- | --- | --- | --- | --- | --- | --- | --- | --- | --- | --- | --- | --- | --- | --- | --- | --- | --- | --- | --- | --- | --- | --- | --- | --- |
|  |  | Total  alleles |  | L223P | Δ229-235 | Δ249-252 | R267W | V331E | E340K | T385A | Combined |  | G35E | T39M | L76fs | V88M | V112I | R131G | A210V | A240E | R244S | A250T | A275V | A301S | R310H | N337D | A341V | Combined |  | **P332Q** ^†^ |
| **Cases** | MinE DF1 | 8,732 |  | 1 | 2 | 1 | - | 1 | 1 | 1 | 7 |  | - | - | - | - | 1 | - | - | 1 | 1 | 1 | 1 | 3 | 1 | 3 | 2 | 14 |  | 107 |
|  | Answer ALS | 1,412 |  | - | 1 | - | 1 | - | - | - | 2 |  | 1 | - | - | - | - | 1 | - | - | - | - | - | 1 | - | - | - | 3 |  | 16 |
|  | MinE DF2+* | 3,176 |  | - | - | - | - | - | - | 1 | 1 |  | - | - | - | - | - | - | - | - | - | - | - | - | 1 | - | - | 1 |  | 40 |
|  | GENESIS | 5,936 |  | - | 2 | - | - | - | - | - | 2 |  | - | - | - | - | - | - | - | - | - | - | - | - | - | - | 1 | 1 |  | 15 |
|  | UCL | 1,366 |  | - | - | - | 1 | - | - | - | 1 |  | - | - | - | - | - | - | - | - | - | - | - | 2 | - | - | - | 2 |  | 5 |
|  | HSP study | 1,612 |  | - | - | - | - | - | - | - | 0 |  | - | - | - | - | - | - | - | - | - | - | - | - | - | - | - | 0 |  | 15 |
|  | Total | **22,234** |  | 1 | 5 | 1 | 2 | 1 | 1 | 2 | **13** |  | 1 | 0 | 0 | 0 | 1 | 1 | 0 | 1 | 1 | 1 | 1 | 6 | 2 | 3 | 3 | **21** |  | 198 |
|  |  |  |  |  |  |  |  |  |  |  |  |  |  |  |  |  |  |  |  |  |  |  |  |  |  |  |  |  |  |  |
| **Controls** | MinE DF1 | 3,664 |  | - | - | - | - | - | - | - | 0 |  | - | 1 | 1 | 1 | - | - | - | - | - | - | - | - | - | 1 | - | 4 |  | 47 |
|  | Answer ALS | 184 |  | - | - | - | - | - | - | - | 0 |  | - | - | - | - | - | - | 1 | - | - | - | - | - | - | - | - | 1 |  | 3 |
|  | MinE DF2+* | 812 |  | - | - | - | - | - | - | - | 0 |  | - | - | - | - | - | - | - | - | - | - | - | - | - | - | 1 | 1 |  | 15 |
|  | GENESIS | 7,330 |  | - | - | - | - | - | - | - | 0 |  | - | - | - | - | - | - | - | - | - | - | 1 | - | - | 1 | 1 | 3 |  | 58 |
|  | UCL | 3,616 |  | - | - | - | - | - | - | - | 0 |  | - | - | - | - | - | - | - | - | - | - | 1 | 1 | 2 | - | - | 4 |  | 38 |
|  | HSP study | 2,490 |  | - | - | - | - | - | - | - | 0 |  | - | - | - | - | - | - | - | - | - | - | - | - | - | - | - | 0 |  | 19 |
|  | LBC | 2,708 |  | - | - | - | - | - | - | - | 0 |  | - | - | - | - | - | - | - | - | - | - | - | - | - | 2 | - | 2 |  | 26 |
|  | Total | **20,804** |  | 0 | 0 | 0 | 0 | 0 | 0 | 0 | **0** |  | 0 | 1 | 1 | 1 | 0 | 0 | 1 | 0 | 0 | 0 | 2 | 1 | 2 | 4 | 2 | **15** |  | 206 |
